# De novo synthesis of fatty acids is regulated by FapR protein in *Exiguobacterium antarcticum* B7, a psychrotrophic bacterium isolated from Antarctica

**DOI:** 10.1186/s13104-016-2250-9

**Published:** 2016-09-20

**Authors:** Rafael A. Baraúna, Diego A. das Graças, Catarina I. P. Nunes, Maria P. C. Schneider, Artur Silva, Marta S. P. Carepo

**Affiliations:** 1 Laboratório de Genômica e Bioinformática, Centro de Genômica e Biologia de Sistemas, Instituto de Ciências Biológicas, Universidade Federal do Pará, Belém, PA 66075-110 Brazil; 2 REQUIMTE/CQFB, Departamento de Química, Faculdade de Ciências e Tecnologia, Campus de Caparica, Universidade Nova de Lisboa, 2829-516 Caparica, Portugal

**Keywords:** Antarctica, Psychrotrophic, FapR, *Exiguobacterium antarcticum*

## Abstract

**Background:**

FapR protein from the psychrotrophic species *Exiguobacterium antarcticum* B7 was expressed and purified, and subsequently evaluated for its capacity to bind to the promoter regions of the *fabH1*-*fabF* and *fapR*-*plsX*-*fabD*-*fabG* operons, using electrophoretic mobility shift assay. The genes that compose these operons encode for enzymes involved in the de novo synthesis of fatty acids molecules. In *Bacillus subtilis*, FapR regulates the expression of these operons, and consequently has influence in the synthesis of long or short-chain fatty acids. To analyze the bacterial cold adaptation, this is an important metabolic pathway because psychrotrophic microrganisms tend to synthesize short and branched-chain unsaturated fatty acids at cold to maintain cell membrane fluidity.

**Results:**

In this work, it was observed that recombinant protein was able to bind to the promoter of the fully amplified *fabH1*-*fabF* and *fapR*-*plsX*-*fabD*-*fabG* operons. However, FapR was unable to bind to the promoter of *fapR*-*plsX*-*fabD*-*fabG* operon when synthesized only up to the protein-binding palindrome 5′-TTAGTACCAGATACTAA-3′, thus showing the importance of the entire promoter sequence for the correct protein-DNA interaction.

**Conclusions:**

Through this observation, we demonstrate that the FapR protein possibly regulates the same operons as described for other species, which emphasizes its importance to cold adaptation process of *E. antarcticum* B7, a psychrotrophic bacterium isolated at Antarctica.

**Electronic supplementary material:**

The online version of this article (doi:10.1186/s13104-016-2250-9) contains supplementary material, which is available to authorized users.

## Background

*Exiguobacterium antarcticum* B7 is a gram-positive, psychrotrophic and free-living bacteria isolated from a biofilm formed in the sediment of Lake Ginger, Antarctic Peninsula. The genome of this strain was obtained by next-generation sequencing [1] and the molecular response to low temperatures was evaluated using both transcriptomic and proteomic approaches [2]. A low temperature influences the expression of several genes and therefore markedly modifies the bacterial metabolism. One of the main molecular modifications observed in *E. antarcitum* B7 cultivated at 0 °C was the differential expression of enzymes that catalyze the de novo fatty acid synthesis [2].

To adapt to cold environments, microorganisms modify the structure of their fatty acid, by decreasing the size of the fatty acid chain and increasing the number of unsaturations in the molecule [3, 4]. Such modifications maintain membrane fluidity in low temperatures. These changes may occur after the synthesis of the fatty acid chain by the DesR–DesK two-component system [5] or during the de novo synthesis of the molecule. In gram-positive bacilli, such as the model bacterium *Bacillus subtilis*, de novo synthesis is performed by the fatty acid synthase II (FASII) system, which is regulated by the FapR regulatory protein [6, 7]. All genes necessary for de novo synthesis of fatty acids including the FapR regulator have been identified in the genome of *E. antarcticum* B7 [1], suggesting a similar mechanism to that observed in *B. subtilis*.

Proteins expressed by psychrotrophic organisms are of great biotechnological interest due to both their important role in acclimation to adverse conditions resulting from decreased temperatures and their practical applications in biotechnological processes [8]. Hence, the FapR regulator of *E. antarcticum* B7 was expressed in *E. coli* BL21 using an ligation-independent cloning (LIC) vector and subsequently purified to validate its function and to demonstrate that this is the main protein in *E. antarcticum* B7 that regulates the fatty acid synthesis regulon during cold adaptation.

## Methods

### DNA extraction and bacterial growth

Cells of *E. antarcticum* B7 were stored in 25 % glycerol until use. Genomic DNA extraction was performed after growth of cultures in 50 ml Tryptic Soy Broth until reaching OD600 0.5. The cells were centrifuged at 8000*g* for 5 min, the supernatant was discarded, and the pelleted cells were used for extraction according to the protocol by Wilson [9].

### PCR conditions and cloning

Primers were designed containing the vector-binding end according to the protocol of the pET-46 Ek/LIC Vector kit (Novagen). The primers used for amplification of the FapR gene was FAPR (5′-GACGACGACAAGATG CGG GTACCTAAAAAAG-3′) and FAPF (5′-GAGGAGAAGCCCGGTTATCTGGACTCCTCCTTAC-3′). Genomic DNA from *E. antarcticum* B7 was used as template for the PCR. The reaction was performed in a total volume of 50 μl and contained 1× MgCl_2_/PCR buffer, 0.2 mM dNTPs, 0.2 μM of each primer and 2 U Taq DNA polymerase high fidelity (Fermentas). Reaction conditions were: initial denaturation step at 94 °C for 2 min; 30 cycles of 94 °C for 1 min, 56 °C for 40 s, and extension at 72 °C for 2 min; and a final extension at 72 °C for 10 min.

Amplicons were purified using the QIAquick Gel Extraction kit (Qiagen), according to the manufacturer’s protocol. Subsequently, amplicons were treated with T4 DNA polymerase and ligated into the pET-46 Ek/LIC vector (Novagen). The plasmids were first transformed by thermal shock into the competent NovaBlue GigaSingles™ cells (Novagen) provided with the expression kit. The selection of clones was performed on selective LB agar medium containing 50 µg ml^−1^ ampicillin. Selected clones were grown in 4 ml of liquid LB medium at 37 °C with shaking at 250 rpm overnight, and the plasmid was extracted using the QIAprep Spin Miniprep kit (Qiagen). Finally, the plasmids containing the target gene were purified and transformed into *E. coli* BL21 for heterologous expression according to the manufacturer’s protocol provided with the competent cells (Novagen).

### Expression assays and protein purification

Initially, expression assays were conducted to determine the best parameters for expression of the target genes. Subsequently, the bacteria were grown in 3 l of medium. First, the bacteria were centrifuged at 8000*g* for 20 min at 4 °C. The supernatant was discarded, and the pelleted cells were suspended in buffer containing 20 mM Tris-HCl, 0.5 M NaCl, 10 mM imidazole, 1 mM protease inhibitor (PMSF) and DNase I. The cells were first frozen at −80 °C for 10 min, thawed at room temperature and subsequently lysed in a FRENCH® Press (Thermo Scientific) four times at a pressure of 20,000 psi. The extract was centrifuged at 8000*g* for 30 min at 4 °C, and the supernatant was ultracentrifuged at 100,000*g* for 90 min at 4 °C. The soluble fraction obtained was stored at −80 °C. The recombinant protein was purified using the HPLC AKTAprime plus system (GE Healthcare). The pET 46 Ek/LIC vector adds a histidine tag to the recombinant protein, and for this reason, the initial purification step for all analyzed proteins utilized a HisTrap HP 5 ml column (GE Healthcare) according to the manufacturer’s protocol. Subsequently, the imidazole present in the protein-containing fraction was removed using a PD-10 desalting column (GE Healthcare). FapR was further purified using the Superdex 200 ×k 26 and Resource Q columns for removal of contaminant proteins. The equilibration buffers used for each purification step are described in the table of the Additional file 1: Table S1.

### Electrophoretic mobility shift assay (EMSA)

Two approaches were used for assaying the binding of the FapR regulatory protein to the promoter of the *fapR*-*plsX*-*fabD*-*fabG* and *fabH1*-*fabF* operons. In the first approach, the oligos of the promoter region were synthesized up to the sequence containing the 17-bp 5′-TTAGTACCAGATACTAA-3′ palindrome identified by Schujman and colleagues [10] as the protein-binding site in the model organism *B. subtilis*. In this approach, the promoter sequence comprising the region from the end of the palindrome to the start of the first gene of the operon was not synthesized. In the second approach, primers were designed to amplify the entire promoter region including approximately 100 bp of the first gene of the operon. The protein-DNA binding reaction occurred for 1 h on ice in buffer containing 10 mM Tris-HCl, 0.2 mM DTT, 0.2 mM EDTA and 0.25 mM KCl. For binding to the DNA of the synthesized promoter, four concentrations of protein were tested (2, 2.8, 3.8 and 4.5 μM). For the amplified region, 2.8 μM of protein was used. After binding, the sample was run on a 7.5 % native PAGE gel for approximately 1 h with O’GeneRuler 1 kb Plus DNA Ladder (Fermentas). Finally, the gel was stained using SYBR® Safe, and the DNA bands were visualized for further analysis.

## Results and discussion

After extraction, the recombinant protein was mainly detected in the soluble fraction of the protein extract (Additional file 2: Figure S1). This soluble fraction was subsequently used for the protein purification steps.

Lipids produced by the FASII system are the main precursors of membrane phospholipids. The composition of these phospholipids directly affects the survival of bacteria at low temperatures [11]. In *E. antarcticum* B7, the synthesis of either saturated or branched-chain fatty acids is coordinated by FAS II system, since all enzymes of this system were found in the genome of *E. antarcticum* B7 [1]. The system responds to the regulatory protein FapR and, therefore, plays an important role in cellular physiology of psychrotrophic organisms. Changes on the membrane composition in the cold, which starts to present higher concentrations of unsaturated and branched-chain fatty acids, has been described in other bacterial species isolated from polar environments such as *Planococcus halocryophilus* Or1 [12].

**Fig. 1:**
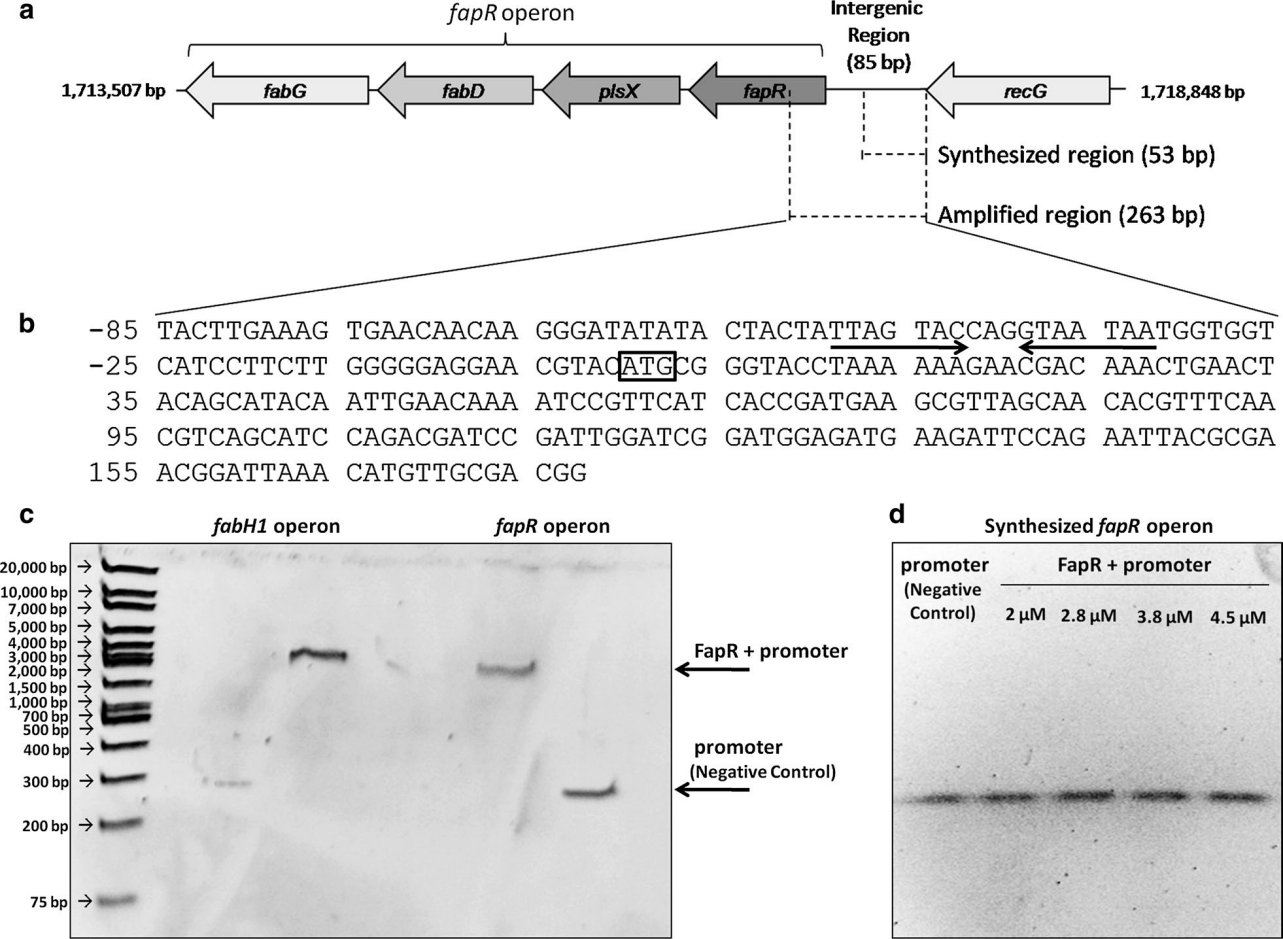
EMSA assay for the FapR protein. **a** Representation of the *fapR*-*plsX*-*fabD*-*fabG* operon region. The genes are represented by *arrows*. The genomic position of the genes is indicated in base pairs at the edge of the figure. The two DNA sequences (amplified and synthesized) used for the EMSA assay are represented by *dotted lines* with their respective sizes. **b** Sequence of the *fapR*-*plsX*-*fabD*-*fabG* promoter amplified by PCR. *Arrows* indicate the 17-bp palindromic sequence, and the start codon of the *fapR* gene is indicated by the *square*. **c** Native PAGE gel of FapR bound to promoters of the *fapR* and *fabH1* operons. The DNA-protein interaction is indicated by the decreased migration of the samples containing the mixture of the regulatory protein with the promoter compared with the negative control (promoter only). (**d**) Native PAGE gel of the FapR protein at different concentrations incubated with the promoter region of the *fapR*-*plsX*-*fabD*-*fabG* operon synthesized only up to the protein-binding palindrome. Regardless of increases in protein concentration, the protein–DNA interaction was not observed

In our study, analysis of the obtained SDS-PAGE gel of the purified protein FapR showed that the protein has a molecular mass of 25 kDa, which is in agreement with the theoretical molecular mass determined of 23.2 kDa without the histidine tag (Additional file 3: Figure S2). The DNA promoter binding of recombinant FapR from *E. antarcticum* B7 was analyzed by EMSA. The protein was able to bind to the promoter region of the two operons (*fapR*-*plsX*-*fabD*-*fabG* operon and *fabH1*-*fabF* operon) amplified by PCR (263-bp amplicon) (Fig. 1a–c). However, the FapR protein was unable to bind to the same promoter region of the *fapR*-*plsX*-*fabD*-*fabG* operon, which was synthesized from the beginning of the intergenic region to the protein-binding palindrome 5′-TTAGTACCAGATACTAA-3′ (53-bp amplicon) identified by Schujman and colleagues [10] as the protein–DNA interaction site (Fig. 1b and d) in *B. subtilis*. This protein-binding palindrome was detected in the promoter sequence of two of the four FapR-regulated operons (Fig. 1b) in *E. antarcticum*. Therefore, the results indicate that despite the importance of the palindromic region for recognition and binding of the regulator, the entire sequence of the promoter region is necessary for the correct interaction between FapR and the promoter region.

Dall’Agnol and colleagues [2] evaluated the gene expression of *E. antarcticum* B7 at 0 °C. In this analysis, the *fabH1* gene which is responsible for the initial reaction of fatty acid synthesis was underexpressed in cold, suggesting a metabolic repression of the entire FASII system at low temperatures. The analysis was performed at the mid-log phase of microbial growth and therefore could describe the changes in the expression of cold acclimation proteins. The downregulation of *fabH1* indicates that the regulatory repressor FapR plays an important role in maintaining the synthesis of new fatty acid molecules at low rates, in order to follow the slow microbial growth in cold. The genes *fabF* and *fabI* were also downregulated at 0 °C, while the operon *fapR*-*plsX*-*fabD-fabG* showed no differential expression.

We found that under standard incubation conditions (the protein-DNA binding reaction was performed on ice), the protein was able to bind to both promoters, indicating that the FapR protein is capable of recognize the promoter sequence and to regulate both operons. This result emphasizes that FapR is possibly the main responsible to maintain the de novo synthesis of branched-chain fatty acids at low rates in cold.

Nevertheless, transcriptional repression at 0 °C of only the *fabH1*-*fabF* and *fabI* operons, as shown by transcriptomic results [2], indicates that could be other regulatory mechanisms involved in the expression of enzymes of the fatty acid biosynthesis. This could explain why the *fabH1*-*fabF* operon had lower expression in the cold, whereas the *fapR*-*plsX*-*fabD*-*fabG* operon showed no differential expression although both operons are regulated by the same protein. For example, Zanphorlin and colleagues [13] demonstrated that GH1 β-glucosidase enzyme of *E. antarcticum* B7 has a different quaternary structure when compared to the homologous enzymes of mesophilic micro-organisms. This differential oligomerization confers high catalytic activity at low temperatures, and is an example of molecular adaptation of *E. antarcticum* B7.

## Conclusions

This study presents the results obtained for the regulatory protein FapR, which complements the previously published data on the mechanisms of cold adaptation of *E. antarcticum* B7 [1, 2]. It became clear that FapR binds to both promoters and possibly regulates the expression of *fabH1*-*fabF* and *fapR*-*plsX*-*fabD*-*fabG* operons. Furthermore, it is apparent that other molecular factors must act to modulate the expression of these operons in the cold since, although both operons are regulated by the same protein, they showed differences in gene expression according to the transcriptome analysis [2].

## Additional files

10.1186/s13104-016-2250-9 Table containing the chemical composition of the buffers used during each protein purification step.
10.1186/s13104-016-2250-9 SDS-PAGE Gel containing the three fractions obtained after the protein extraction protocol. The recombinant protein was mainly detected in the soluble fraction near the molecular weight of 25 kDa, which is in accordance with its calculated molecular weight.
10.1186/s13104-016-2250-9 Gel image showing the purification steps of FapR. Each well of the gel contains the same sample after successive steps of purification using different chromatographic columns. The molecular weight marker is show on the left side of the gel. HisT = HisTrap HP column; Spx200 = Superdex 200 xk 26 column; ResQ = Resource Q column.

## Abbreviations

EDTA: ethylenediamine tetraacetic acid
EMSA: electrophoretic mobility shift assay
FASII: fatty acid synthase II
LIC: ligation-independent cloning
OD600: optical density at 600 nm
PAGE: native polyacrylamide gel electrophoresis
SDS-PAGE: denaturing polyacrylamide gel electrophoresis
PMSF: phenylmethylsulfonyl fluoride
TSB: Tryptic Soy Broth
