# Supplementary material for: De novo synthesis of fatty acids is regulated by FapR protein in Exiguobacterium antarcticum B7, a psychrotrophic bacterium isolated from Antarctica
Source: BMC Res Notes. 2016 Sep 20;9:447. doi: 10.1186/s13104-016-2250-9 (PMC5028935; doi:10.1186/s13104-016-2250-9)
Supplement: Supplementary file 1 — 10.1186/s13104-016-2250-9 Table containing the chemical composition of the buffers used during each protein purification step. [file 13104_2016_2250_MOESM1_ESM.pdf]

Table S1. Buffers used during each protein purification step.

Protein	Extraction buffer	HisTrap HP buffers		Superdex 200 Xk26 buffer	Resource Q buffer
		Ligation	Elution 2		
**FapR**	Tris-HCl 20 mM, NaCl 500 mM, Imidazol 10 mM, protease inhibitor (PMSF) 1 mM and DNase I 1 mM.	Imidazol 30 mM	Imidazol 500 mM	Tris-HCl 50 mM, NaCl 150 mM, pH7,6	Ionic strength gradient of 20 mM to 600 mM of Tris-HCl pH 7.6
