# Supplementary figures and images for: De novo synthesis of fatty acids is regulated by FapR protein in Exiguobacterium antarcticum B7, a psychrotrophic bacterium isolated from Antarctica
Source: BMC Res Notes. 2016 Sep 20;9:447. doi: 10.1186/s13104-016-2250-9 (PMC5028935; doi:10.1186/s13104-016-2250-9)

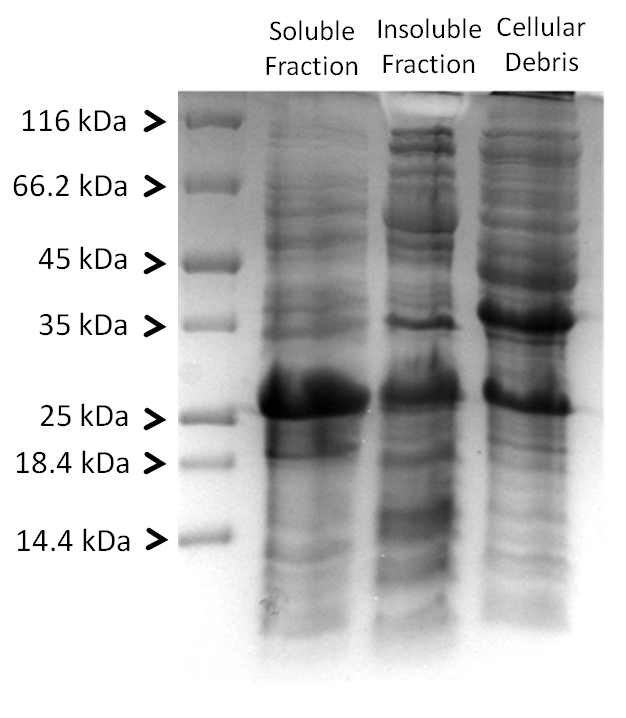

Supplement: Supplementary file 2 — 10.1186/s13104-016-2250-9 SDS-PAGE Gel containing the three fractions obtained after the protein extraction protocol. The recombinant protein was mainly detected in the soluble fraction near the molecular weight of 25 kDa, which is in accordance with its calculated molecular weight. [file 13104_2016_2250_MOESM2_ESM.jpg]

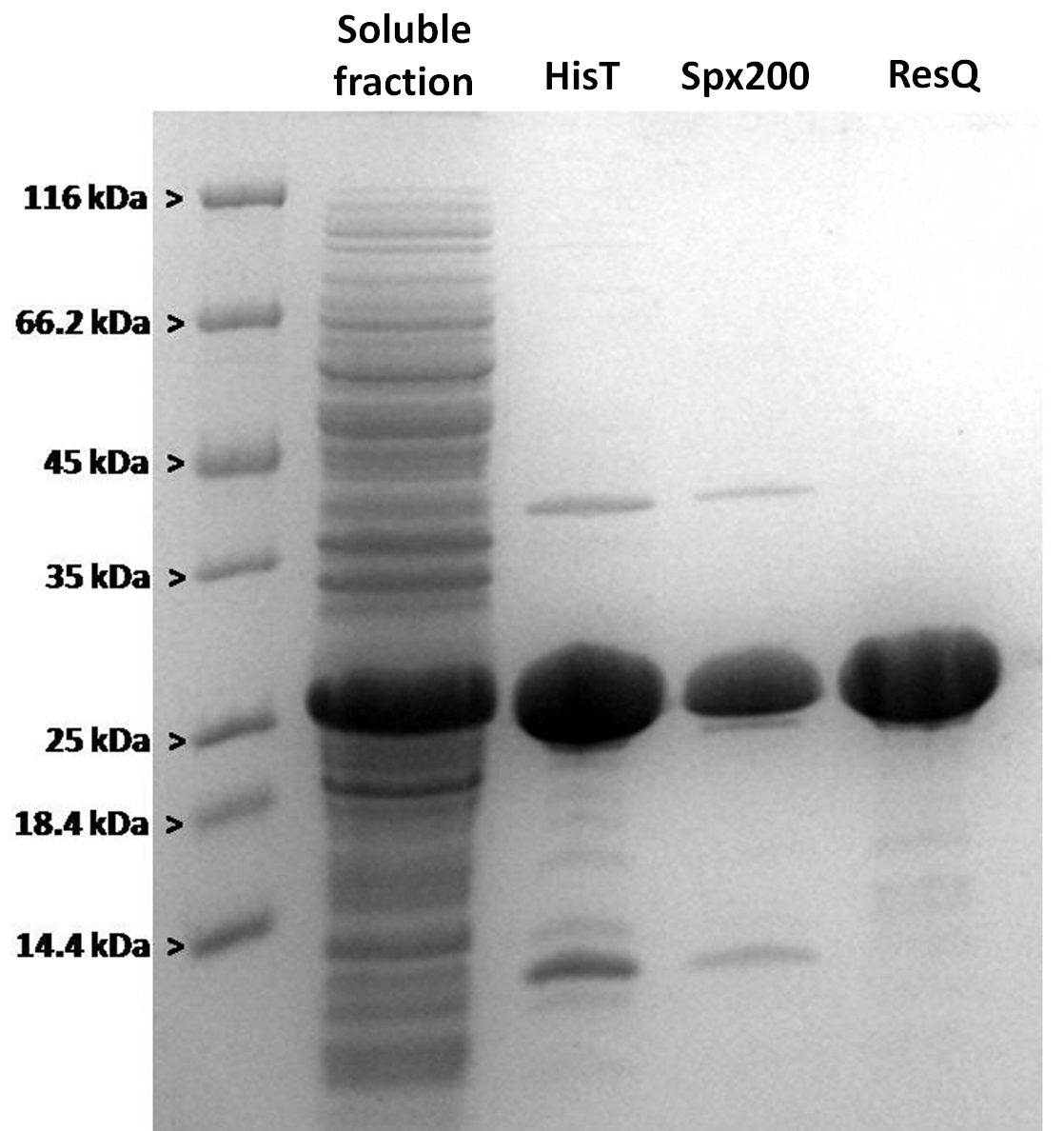

Supplement: Supplementary file 3 — 10.1186/s13104-016-2250-9 Gel image showing the purification steps of FapR. Each well of the gel contains the same sample after successive steps of purification using different chromatographic columns. The molecular weight marker is show on the left side of the gel. HisT = HisTrap HP column; Spx200 = Superdex 200 xk 26 column; ResQ = Resource Q column. [file 13104_2016_2250_MOESM3_ESM.jpg]
